# Taste and Smell Alterations (TSAs) in Cancer Patients

**DOI:** 10.3390/diseases12060130

**Published:** 2024-06-19

**Authors:** Davide Rosati, Pierluigi Mastino, Martina Romeo, Giulia de Soccio, Daniele Pentangelo, Carla Petrella, Christian Barbato, Antonio Minni

**Affiliations:** 1 Division of Otolaryngology-Head and Neck Surgery, Ospedale San Camillo de Lellis, ASL Rieti-Sapienza University, Viale Kennedy, 02100 Rieti, Italy; davide.rosati@asl.rieti.it (D.R.); pierluigi.mastino@gmail.com (P.M.); m.romeo@asl.rieti.it (M.R.);; 2 Institute of Biochemistry and Cell Biology (IBBC), National Research Council (CNR), Sapienza University of Rome, Policlinico Umberto I, Viale del Policlinico 155, 00161 Roma, Italy; carla.petrella@cnr.it; 3 Department of Sense Organs DOS, Sapienza University of Rome, Viale del Policlinico 155, 00161 Roma, Italy

**Keywords:** taste, smell, taste and smell abnormalities (TSAs), head and neck cancer

## Abstract

Recently, smell and taste disorders have seen renewed interest, as these symptoms are frequent complications of SARS-CoV-2 infection, since approximately 60% of patients affected by COVID-19 have shown olfactory and gustatory alterations. Otolaryngology pays attention to taste and smell abnormalities (TSAs), especially when associated with oncology. TSAs are common symptoms in people affected by cancer, yet they are ignored and underestimated. The clinical outcome of TSAs in cancer evidences the importance of identifying them with chemotherapy or radiotherapy in general, and they are associated with many types of cancer. We recognize the findings of the literature on TSAs in cancer, evaluating how it is important to consider and identify these disorders concerning reduced food enjoyment or inappropriate nutrient intake, and modulating the nutritional status, quality of life, and impact of therapy. This review aims to critically evaluate and recognize the assessment and clinical perspectives of taste and smell disorders in a cancer population.

## 1. Introduction

The human chemical senses of taste and smell are essential to life. Smell is a powerful sense. not only for detecting and identifying odors but also for protecting us from environmental events, the recognition of the edibility of food, and, in a special way, for social interaction and emotional life, by modulating our mood, thoughts, behavior, and cognition. It is also essential for defining taste, identifying volatile aroma molecules, and completing the taste sensation. Smell disorders range from hyposmia, a reduced sense of smell, to anosmia, including incidental changes in the normal sense of smell, referred to as parosmia [1]. Recently, new interest was directed to taste and smell disorders, as these symptoms are frequent complications of SARS-CoV-2 infection; approximately 60% of COVID-19 patients experienced olfactory and gustatory dysfunctions [2]. The literature describes five basic tastes: sweet, sour, bitter, salty, and umami [3]. The complete lack of taste is ageusia, while the decreased sensitivity to taste is called hypogeusia. An excessive sensitivity to taste is hypergeusia, while phantageusia is the perception of an unpleasant taste without an appropriate gustatory stimulus. The sense of taste begins with the activation of taste receptor cells. Taste receptor cells are epithelial cells capable of receiving and retransmitting gustatory, olfactory, and trigeminal nervous stimuli [4]. Odor coding occurs via olfactory circuits consisting of approximately 400 peripheral olfactory receptors, approximately 10^7^ olfactory sensory neurons, and central structures, via the ortho-nasal and the retro-nasal route. Four categories of smell disorders are classified: anosmia (absence of smell perception), hyposmia (reduced smell perception), parosmia (impairment of a commonly detected smell), and phantosmia (perception of odors in their absence) [1,5,6,7]. Taste and smell abnormalities (TSAs) are frequently reported in solid and hematological tumors [8]. TSA is a common symptom in cancer patients, yet it is often ignored and underestimated. The reported prevalence ranges from 20% to 86% for taste alterations [9] and 5% to 60% for smell alterations [10]. TSAs may also contribute to increased risk of malnutrition, mood modifications, and reduced social interaction and quality of life [11]. In addition, they may be a factor in anorexia-cachexia cancer syndrome [12], by significantly influencing nutritional status [13]. The clinical consequences of TSA in cancer highlight the importance of identifying and managing these symptoms, as reflected in the literature on TSA related to chemotherapy (CT) or radiotherapy (RT), especially of the head and neck [14,15]. According to these studies, patients undergoing head and neck radiation therapy have a prevalence of TSA of 50–70%, but estimates vary widely among patients undergoing chemotherapy (15–70%). Indeed, there is consensus on the TSA underestimation in cancer [16]. It is important to consider and identify TSA, as it can lead to a reduction in nutrition and an unbalanced intake of nutrients, with a profound impact on nutritional status, quality of life, and therapeutic efficacy. This review aims to critically evaluate and recognize the assessment, prevalence, and clinical perspectives of taste and smell disorders in a cancer population.

## 2. Materials and Methods

The search was performed in November 2023 in PubMed, Web of Science and Embase. The search terms used are the following: “olfactory nerve” AND “chemotherapy”, “loss of smell” AND “chemotherapy”,“loss of smell” AND “cancer”,“loss of smell” AND “head and neck cancer”,“taste smell disorders” AND “head and neck cancer”, “taste smell disorders” AND “cancer”, “taste smell disorders” AND “chemotherapy”, “taste smell disorders” AND “radiotherapy”, “anosmia” AND “chemotherapy”, “anosmia” AND “cancer”. We critically evaluate research published in the last 10 years (from 2013 to 2023) which measured taste and smell function and assessed the status of appetite and dietary intake. We included studies with objective measures of taste and smell function, rather than subjective or self-reported changes in taste and smell. The rationale for this selection was based on the observation that patients often do not distinguish between taste and smell or confuse sensory constructs with hedonic constructs, such as appetite or enjoyment of food [17]. Articles were included if they were available in full text and the English language. Non-cancer diagnosis studies and pediatric studies were excluded. We started from 672 articles that could be included. After a rapid evaluation of contents, we selected 126 articles that could satisfy the parameters; we selected reviews, and we excluded articles published before 2013; in the end, we found 33 articles that matched all the inclusion criteria. This review tried to critically discover a correlation between the current trajectory of an oncological patient and taste and smell disorders, providing a thorough evaluation of the literature about the prevalence and clinical consequences of these disorders. The aim was to examine the current literature, focusing on the last 10 years, to understand if there are recent recommendations or new routes for developing prevention strategies for taste and smell alterations in oncological patients.

## 3. Results

### 3.1. Assessment of TSAs in Cancer

When analyzing changes in taste and smell, we evaluate detection, which is the awareness of a sensation of taste or smell, and recognition, which indicates that a taste or smell can be recognized and named [18]. Examining the literature, objective taste assessment methods used were electrogustometry, liquid tastants, and filter paper discs/strips, though, as underlined by Epstein [19], each method has some limitations. Regarding smell, the objective methods to assess it were “Sniffin sticks”, inhalation of solutions, and the “University of Pennsylvania Smell Identification Test” (UPSIT), but even in this case, there is no certainty as to which objective assessment method can be recommended [20]. An estimate of the true prevalence of taste and smell disorders is complex, given variations in methodology and confounding factors such as the use of antiemetic and analgesic drugs and the frequent use of subjective and objective detection methods. Moreover, as underlined by various authors [21,22], TSAs are under-recognized by medical oncologists in one-third of cases, or under-contemplated, with these symptoms considered untreatable. On the other hand, patients may be aware of these problems but might be unable to articulate their taste and smell alterations. All these situations may exacerbate the under-recognition of TSAs. In any case, even if the gustatory and olfactory sensory pathways are anatomically separate, they are closely connected in relation to the processing of the perception of food, suggesting that the two senses should be evaluated together [23]. In addition, the optimal performance of the olfactory function is between 20 and 60 years old [24] (Table 1).

### 3.2. Prevalence of TSAs in Cancer

Several studies suggest that the main reasons causing the deterioration of smell are linked to factors associated with age, diabetes, hypertension, or repeated infections of the olfactory epithelium [50,51]. On the other hand, with age, the loss of taste is much less pronounced the loss of than smell [52]. Furthermore, some drugs, such as antihypertensives, diuretics, or antidepressants, could cause abnormalities in the sensation of taste and smell, in addition to xerostomia. Other predisposing conditions are oral infections, smoking, alcohol abuse, and chronic rhinosinusitis [53]. An interesting study by Dhuibhir et al. shows a high prevalence of taste and smell alterations in newly diagnosed cancer patients before treatment [38]. Taste and smell alterations are very disabling side effects in oncologic patients, impacting the daily lives of cancer patients [39,54,55]. Other studies demonstrate the fact that TSAs during chemotherapy have a strong impact on the daily life of patients; these studies hypothesize that these patients have a reduced quality of life, suffer weight loss and nutritional deficiencies, and obtain unfavorable therapeutic outcomes [33,42,43,44,56,57,58]. In the literature, focusing on oncological patients who underwent chemotherapy, the incidence of smell alterations has been reported to be between 16% and 49%, while taste alterations have been reported to be between 20% and 70% [31,48]. This difference could be explained by the difference in the turnover rate of smell and taste receptors (mean 30 days versus 10 days) or by the hypothesis that the olfactory epithelium is less susceptible to damage. For many cancer patients, CT is the primary form of treatment, and its short- and long-term effect on chemosensory alterations is less understood. Radiotherapy is a well-known cause of chemosensory dysfunction, as it can lead to direct damage of taste receptors, synaptic uncoupling, and other neurological damage [59,60]. RT (especially in head and neck cancer) can also cause hypo-salivation and dry mouth, resulting in reduced delivery of chemical stimulants to receptors, resulting in a prevalence of 50% of smell and 70% of taste alterations [61,62]. In the literature, taste alterations in cancer patients undergoing RT are quite well described, suggesting that the minimum radiation dose able to cause taste alterations is 15–30 gray [63,64], while there is no clear consensus on whether smell alterations occur during RT. No significant differences have been described between the use of conventional and hyper-fractioned RT [65], although parotid-sparing IMRT has been related to an improved food intake after treatment [66]. On the other hand, in the literature, there are no studies aimed at characterizing the severity of TSAs during RT. Exploring the area of hormone therapy and immunotherapy makes it possible to extrapolate data about their impact on TSAs, even if some previous studies suggest the association between impaired smell and congenital or post-menopausal hypogonadism [67,68].

### 3.3. Prevalence of TSAs in Head and Neck Cancer

A specific focus must be placed on head and neck cancer (HNC), in which the primary tumor and the consequent treatments often contribute together to affect nasal, oral, and pharyngeal functions that impact oral intake [34,40,45,69]. Unlike other types of cancer, sensory alterations in HNC patients are caused by the primary tumor and treatments, although the mechanisms are not known. In irradiated patients, after evaluation with subjective and objective methods, taste alterations were observed in 96% and 79% [69]. In HNC, dysgeusia may begin with mucosal damage due to cytotoxicity and neurotoxicity resulting from RT and drug administration. CT may have cytotoxic effects on taste and smell through systemic effects, in addition to driving a reduced salivary secretion of the gingival crevice [36,70]. In HNC patients, surgery in the oral cavity can even lead to taste alterations through the surgical damage of chemosensory nerves [71]. An interesting review [72] indicates that olfactory perception of foods among head-and-neck-cancer patients is more frequent than changes in taste perception, as are changes in texture, temperature, and other oral sensations, such as hotness and cold. In these patients, smell is less affected by RT than taste, and can recover within 6 to 9 months after RT [73]. Another aspect of TSA is related to paraneoplastic syndrome, which includes tumor-induced alterations in the production of hormones, growth factors, and antigen–antibody complexes. Olfactory dysfunction is part of the paraneoplastic syndrome of malignant breast and lung neoplasms [30,46,47,74,75]. It may be due to HNC-related production of antibodies against neural epitopes, such as the retinopathy-associated anti-recoverin antibody, and inhalation of tumor-related toxicants produced in the airways [76,77]. Another interesting recent study [25] highlights the fact that olfactory disorders can be detected with tests in more than 90% of patients treated with head and neck cancer, even if self-reported olfactory perception was normal. Moreover, approximately 20% of these patients have a total loss of smell. Furthermore, the researchers propose to test the hypothesis of considering olfactory tests as a screening method to detect HNC early in patients with risk factors such as smoking, alcoholism, age, and gender.

## 4. Discussion

The etiology of TSAs, probably due to the heterogeneity of studies on tumor populations and the multifactorial nature of taste and smell abnormalities, has not yet been clarified. The hypothesis that seeks to explain the biological basis of drug-induced taste disorders highlights the following: the probable interaction with the taste receptors located on the apical side of the tongue; genetic differences that influence taste perception; the taste sensations caused by injectable drugs; and drug interactions caused by the combined use of multiple drugs. The most accredited theory is based on cytotoxic damage to the rapidly dividing cells such as olfactory- and gustatory-receptor and mucosal cells [19,78,79,80] as well as the alteration of saliva and mucus production; it can affect taste through the development of oral mucositis, dry mouth, and dental caries. Cytotoxic drugs can also exhibit independent effects on taste and smell by inducing a specific odor or even affecting the central or peripheral nervous system [81]. The chemotherapeutic agents that act most on TSA are docetaxel, carboplatin, anthracycline, paclitaxel, and vinorelbine. However, as reported in the literature, the effect of different chemotherapy treatments on the severity of specific taste and smell alterations is not yet known, and wide gaps in the research regarding TSAs in different patient populations are present [26]. Another study described the fact that changes in smell threshold after CT were significantly more intense with 5-fluorouracil and capecitabine if compared to cisplatin and carboplatin [82]. However, TSAs are sometimes present even before the beginning of anticancer treatments, suggesting other mechanisms are involved. For example, some authors have highlighted factors that modify the increase in the inflammatory state, changes in carbohydrate and lipid metabolism, modification of the oral microbiota, mucositis, zinc depletion, and changes in the quantity and composition of saliva, which are important to evaluate [83]. To date, it has been shown that taste and smell alterations are often largely transient, and usually recover within the first 3–6 months after the end of chemotherapy [84,85,86]. A recent review of chemotherapy drugs and taste/smell alterations [87] affirmed that Docetaxel, Paclitaxel, Nab-Paclitaxel, Capecitabine, Cyclophosphamide, Epirubicin, Anthracyclines and 5-FU analogs are the drugs most frequently associated with taste and smell disorders. Moreover, as demonstrated by Gamper et al. [88], they underlined the fact that dysgeusia and dysosmia were strictly associated with breast, uterus, and colorectal cancer. Other evidence found in the literature is represented by the higher incidence of salty and sour taste alteration during cancer treatments [41,89] and the number of chemotherapy cycles, gender, metastases, and the primary tumor’s location in the uterus, testicles or head and neck, as important risk factors for taste and smell disorders [32]. Other studies underlined the significant worst outcome in terms of recovery of smell and taste in patients who underwent trastuzumab treatment for breast cancer [35] or the significative prevalence of reduced taste and smell of food in prostate cancer treatment receiving hormone therapy and/or chemotherapy, leading to reduced appetite and ≥10% weight loss [27]. Many studies have attempted to systematize the ways of supporting, preventing, and treating chemosensory alterations in cancer patients [90], without the possibility of generalizing a model. Precision medicine could be an opportunity to respond to this clinical problem. However, the current literature contains few univocal recommendations because of the variability of perception of these disorders in cancer patients, the wide heterogeneity of the population involved, the frequent underestimation of the problem among healthcare workers, and the difficulty of standardizing the diagnostic and preventive modalities in cancer care. Currently, taste and smell alterations can be assessed through clinical methods (objective) or self-reported methods (subjective). Therefore, there is also a chronic bias of problematic standardization of results due to the combination of different measurement methods and experimental designs. Multiple objective measures were used to assess taste and smell perception [91] leading to a mix of positive and negative results, often in contradiction with each other [92,93,94]. Interestingly, few patients spontaneously report symptoms in taste and smell, and are often overlooked by oncologists [95]. All these observations suggest that the lack of generalizability of selected interventions remains the greatest limitation [96]. All these studies suggest the development of standardized methods to ensure that TSAs associated with sensory changes are quantitative and reliable for use by clinicians and researchers. Lastly, a recent study evidenced the adverse drug reactions (ADRs) using the FDA Adverse Events Reporting System (FAERS) focused on olfactory and gustatory dysfunction [97]. It analyzed the FAERS database from 2011 to 2021, searching for “anosmia, hyposmia, olfactory test abnormal, olfactory nerve disorder, hallucination olfactory, parosmia, ageusia, hypogeusia, dysgeusia, and taste disorder” reported in ADRs related to OGD. Interestingly, anticancer and immunomodulating drugs were frequently associated with general gustatory dysfunction, accounting for 36% of the reports and 22.9% for smell dysfunction, suggesting that these molecules are the most frequently reported for TSA adverse drug reactions.

## 5. Conclusions

Alterations in taste and smell can contribute to malnutrition, representing an important predictor of morbidity and mortality, and evaluating a response to treatment toxicity in cancer [28,45]. A standardized and systematic study of taste and smell behavior in cancer patients before, during, and after treatments seems very important for its practical implications on oncological outcomes [46]. A clearer understanding of the effect of taste and smell alterations on elements of eating behavior can support the design and testing of clinical strategies aimed at modulating unwanted effects on chemosensory function caused by cancer treatment [40,47]. Few studies focused on taste and smell disorders in cancer patients, dividing the population into treatment-naive patients, those undergoing RT, CT, RT + CT, hormone therapy, and immunotherapy, and those who have completed treatment and have long survival [29,34,37,49]. Moreover, we noticed a wide heterogeneity of the evaluation tools used and the fact that no gold-standard assessment tool has been identified; this does not allow for an accurate generalizability of the results. Therefore, it seems very difficult to standardize the evaluation, and new research is needed to explore, standardize, and try to manage the oncological patient’s overall experience of taste-and-smell treatment [40,46,47]. In conclusion, this scoping review has identified gaps in the current literature and topics for future research in this field. Observational studies were identified for the following: to determine “risk factors” for TSAs (i.e., type of cancer, age, BMI, and performance status); to determine the precise etiology of TSA disturbance; to determine the etiology of different subtypes and gradation of TSAs (i.e., ageusia and dysgeusia and anosmia and hyposmia); to focus on patient care, to understand the needs and priorities for management and therapy; and for the development and validation of a specific taste-and-smell assessment tool for this group of patients.

## Figures and Tables

**Table 1:** Studies discussed in this report.

Reference	Treatment	Objective	Alterations Studied	Type of Cancer	Year of Publication
Da Silva [25]	Naive patients	Compare oncological-naive patients and healthy controls	Smell	All cancers except for those involving nose, anterior cranial fossa or brain regions near primary olfactory structures	2023
Amézaga [26]	Chemotherapy and/or hormone therapy	Investigate TSAs in patients undergoing treatment	Taste and Smell	Prostate cancer	2018
Alonzi [27]	Chemotherapy and/or hormone therapy	Investigate TSAs in patients undergoing treatment	Taste and Smell	Prostate cancer	2021
Campagna [28]	Chemoterapy	Investigate taste alterations across chemotherapy regimens	Taste	primary cancers	2018
Leyrer [29]	Radiotherapy	Investigate TSAs in patients undergoing treatment	Taste and Smell	Brain and nasopharynx cancer	2014
Denda [30]	Chemoterapy	Investigate taste alterations in patients undergoing treatment	Taste	Breast cancer	2020
Kaizu [31]	Chemoterapy	Investigate taste alterations across chemotherapy regimens	Taste	Various primary cancers	2021
Malta [32]	Chemotherapy	Investigate taste alterations across chemotherapy regimens, localization of primary tumor and patient features	Taste	Various primary cancers	2022
Sicchieri [33]	Chemotherapy	Investigate taste alterations in patients undergoing treatment	Taste	Various primary cancers	2019
Lilja [34]	Surgery, microvascular free-tissue transfer reconstruction, radiotherapy	Investigate TSAs in patients before, during and after treatment	Taste and Smell	Head and Neck cancer	2018
De Vries [35]	Chemotherapy and/or immunotherapy	Compare patients undergoing treatments with healthy controls	Taste and Smell	Breast cancer	2018
Epstein [36]	Chemotherapy, radiotherapy	Investigate TSAs in patients undergoing treatment	Taste and Smell	Head and Neck cancer	2020
de Vries [37]	Chemotherapy	Investigate TSAs in patients undergoing treatment	Taste and Smell	Oesophagogastric cancer	2019
Dhuibhir [38]	Naive patients	Investigate TSAs in oncological patients before treatments	Taste and Smell	Solid tumors	2020
McGettigan [39]	Various treatments	Investigate TSAs in patients with advanced cancer	Taste and Smell	Advanced cancer (various primary cancers)	2019
Alvarez-Camacho [40]	Surgery, radiotherapy or chemotherapy	Investigate TSAs in patients before, during and after treatment	Taste and Smell	Head and Neck cancer	2016
Drareni [41]	Chemotherapy	Investigate TSAs in patients undergoing treatment	Taste and Smell	Lung cancer	2023
Jeong [42]	Chemoterapy	Investigate TSAs in patients undergoing treatment	Taste and Smell	Breast cancer	2023
Hannon [43]	Various treatments	Investigate TSAs in patients with advanced cancer	Taste and Smell	Advanced cancer (various primary cancers)	2023
Hiroyuki [44]	Various treatments	Investigate the impact of TSAs on dietary intakes and cachexia-related quality of life	Taste and Smell	Advanced cancer (various primary cancers)	2023
Riva [45]	Surgery	Compare laryngectomized long-term survivors with control subjects	Taste and Smell	Laryngeal carcinoma	2017
McGreevy [46]	Surgery, radiotherapy or chemotherapy	Investigate TSAs in patients undergoing treatment	Taste and Smell	Lung cancer	2014
Belqaid [47]	Surgery, radiotherapy or chemotherapy	Investigate TSAs in patients undergoing treatment	Taste and Smell	Lung cancer	2016
Spotten [48]	Naïve patients	Investigate TSAs in oncological patients before treatment	Taste and Smell	Solid tumors except head and neck	2016
Walliczek-Dworschak [49]	Chemotherapy	Investigate TSAs in patients undergoing treatment	Taste and Smell	Testicular cancer	2017
Barbosa [25]	Naive patients	Compare oncological-naive patients with healthy controls	Smell	All cancers except for those involving nose, anterior cranial fossa or brain regions near primary olfactory structures	2023
